# Vitamin D Concentration Changes after Bariatric Surgery

**DOI:** 10.1155/2023/4828052

**Published:** 2023-09-20

**Authors:** Vanessa Mayana Alves Baad, Narriane Chaves Pereira de Holanda, Juliana Fonseca Nogueira Alves, Francisco Bandeira, Ana Célia Oliveira dos Santos, Amanda Alves Marcelino da Silva, Taisy Cinthia Ferro Cavalcante

**Affiliations:** ^1^Faculty of Medical Sciences, University of Pernambuco, Recife, Pernambuco, Brazil; ^2^Department of Endocrinology, Federal University of Paraiba, João Pessoa, Paraíba, Brazil; ^3^Department of Nutrition, University of Pernambuco, Petrolina, Pernambuco, Brazil

## Abstract

**Introduction:**

Bariatric surgery causes physiological and anatomical changes in the gastrointestinal tract that interfere with intestinal absorption and, consequently, with the nutritional status, especially about vitamin D. The aim of the study was to evaluate the vitamin D levels and body composition of these patients in the Roux-en-Y gastric bypass (RYGB) and vertical sleeve gastrectomy (VSG) types of surgery.

**Methods:**

The retrospective cohort study included a population of 120 patients aged between 18 and 65 years, with class II or III obesity, undergoing bariatric surgery procedures (VSG or RYGB-type). Data were collected on the degree of obesity, age, average time since surgery, and gender. The individuals underwent a complete physical examination, measuring blood pressure, weight, height, waist, and neck circumference. In addition to calculating the percentage of loss of body weight and assessing body fat, the food frequency and physical activity of these patients were evaluated. Blood was collected, and the insulin variables, hydroxyvitamin D (25OHD), were analyzed.

**Results:**

There was a significant difference between groups only for PTH, total BMD, and insulin variables. A significant intragroup difference was found in the variables' body mass index (BMI) and vitamin D for the vertical sleeve gastrectomy group and BMI for the RYGB group.

**Conclusion:**

The analysis between the groups of procedures, similarity in body composition and postsurgical vitamin D levels, with significant differences only for PTH, BMD, and insulin variables, demonstrates that both procedures are effective in reducing fat mass.

## 1. Introduction

With the increasing prevalence and high impact on mortality and morbidity due to the high number of metabolic complications, obesity is considered a serious public health problem, reaching epidemic proportions in most countries [1, 2]. Bariatric surgery is a common and effective method for treating severe obesity, and Roux-en-Y gastric bypass (RYGB) and vertical sleeve gastrectomy (VSG) types of surgery are associated with rapid weight loss and reduced comorbidities in obese individuals [3]. VSG-type bariatric surgery is a restrictive treatment that anatomically alters the gastrointestinal tract to promote a decrease in food intake, leading, after surgery, to a reduction in the absorption of lipids and other nutrients. Roux-en-Y gastric bypass (RYGB) combines the classification of restrictive and malabsorptive surgery, also culminating in postsurgical absorption limitation [4].

These structural and physiological changes in the gastrointestinal tract interfere with intestinal absorption and, consequently, with nutritional status, especially in terms of calcium and vitamin D, which are considered essential in the hormonal regulation of the parathyroid (PTH) [5]. It is common for these changes to lead to macronutrient and micronutrient deficiencies, and patients often develop diseases such as anemia, osteoporosis, and protein deficiency. Even before the surgical procedure, most of these obese patients are already in nutritional deficit, with vitamin D and iron deficiency being the most common [6]. As a result of the surgery, a decrease in the levels of heat shock proteins in the jejunal mucosa, as coactivators of the vitamin D receptor, was reported, causing calcium absorption and bone metabolism to be impaired [7].

Studies describe a relationship between fat mass and total 25-hydroxyvitamin (25(OH)D) stores in obese subjects undergoing bariatric surgery and healthy controls [8, 9]. Since obesity has been associated with lower serum levels of 25(OH)D, which is a consistent finding among adults and children of different ethnicities and in the most varied geographic locations, this relationship between vitamin D levels and obesity raises several questions, such as, is vitamin D deficiency a cause or consequence of obesity? If it is a consequence, what leads obese individuals to develop this deficiency? What are the clinical consequences of this relationship? What happens to vitamin D levels with the rapid postsurgical weight change? [10]. In this study, total stores were higher in the group of obese patients who underwent surgery, with no difference in serum concentrations of 25(OH)D. The results reinforce the hypothesis of volumetric dilution of vitamin D in adipose tissue found in obese individuals [8, 9]. However, there are still gaps about its association with other body composition markers, such as muscle mass levels and biomarkers associated with 25-hydroxyvitamin D.

The present study was carried out considering the relevance of the subject and knowledge gaps regarding the behavior of biomarkers associated with vitamin D deficiency in relation to the body composition of obese patients undergoing bariatric surgery. The aim of the study was to evaluate the vitamin D levels and body composition of these patients in Roux-en-Y gastric bypass (RYGB) and vertical sleeve gastrectomy (VSG) types of surgery.

## 2. Methods

A retrospective cohort study was developed, whose study population included 120 patients between 18 and 65 years old, with at least class II (≥35 kg/m^2^) or class III (≥40 kg/m^2^) obesity associated with comorbidities, submitted to a bariatric surgery procedure (vertical sleeve gastrectomy type or Roux-en-Y gastric bypass). Informed consent was signed by all subjects before the tests. The procedures were approved by the Research Ethics Committee of the University of Pernambuco, Recife, PE, Brazil (Number 4.195.468), and were in agreement with the 466/2012 resolution of the National Health Council of 12 December 2012. The degree of obesity was defined by calculating the body mass index (BMI), resulting from weight (kg)/height (m^2^). The average age of patients who underwent the VSG bariatric surgery procedure was 41.74 ± 0.92 (*n* = 57), while for the Roux-en-Y gastric bypass (RYGB) procedure, it was 41.92 ± 0.84 (*n* = 60). The average times these patients had undergone the surgical procedures were between 1 and 3.9 years (2.21 ± 0.16 for VSG and 1.79 ± 0.21 for RYGB) and over 4 years (6.26 ± 0.79 for VSG and 8.08 ± 0.60 for RYGB). The male population represented 7.14% (*n* = 4) of the total for the VSG procedure, while the female population represented 92.86% (*n* = 56). For the RYGB procedure, the male population represented 10% (*n* = 6) of the total, while the female population represented 90% (*n* = 54). In total, for both procedures, the male population represented 8.33% (*n* = 10) of all participants, while the female population represented 91.66% (*n* = 110). The collection of these data is shown in a table, available for consultation in Appendix A. The inclusion criteria for the study were as follows: having class II or III obesity, having undergone bariatric procedures, and having one or more associated comorbidities. Exclusion criteria were having menopause, end-stage chronic kidney disease (CKD), and chronic use of corticosteroids (prednisone or equivalents greater than 5 mg/day) or antiobesity drugs.

After signing the free and informed consent form (FICF), the patient answered a specific questionnaire and underwent a complete physical examination. Measurements of weight, height, waist circumference, and neck circumference were taken at the first visit, according to the recommendations and classification of the World Health Organization (WHO) [11, 12]. The percentage of total body weight loss (%TWL) was calculated to assess weight change after the bariatric procedure. Body fat (fat mass in kg and body fat percentage) was assessed using dual-energy X-ray absorptiometry (DXA).

The assessment of food frequency was performed using the Food Consumption Frequency Questionnaire (FFQ) [13]. The International Physical Activity Questionnaire (IPAQ) was used to assess physical activity [14]. The measurement of systemic blood pressure was performed according to the recommendations of the current American guideline [15].

After overnight fasting, blood was collected by using the vacuum collection system. For 25-hydroxyvitamin D, insulin, and leptin, serum was collected in tubes with a gel separator. After collection, the tubes were homogenized by the inversion method 8 times and kept in a vertical position for 30 minutes for clot retraction. After 30 minutes, the tubes were centrifuged at a rotation of 3,000 revolutions per minute (RPM) for 10 minutes. After centrifugation, the measurements were performed. For glucose quantification, plasma was collected using a tube with sodium fluoride + EDTA. After collection, the sample was centrifuged at 3,000 RPM for 10 minutes and the dosage was administered.

Serum insulin and 25-hydroxyvitamin D were measured using a competitive chemiluminescent immunoassay (DX1, Beckman Coulter, Brea, CA, USA). It is of great importance to assess vitamin D with insulin and insulin resistance. Studies show that the effects can also be direct (via vitamin D stimulation of insulin receptor expression, thus increasing the insulin response to glucose stimulation) or indirect (via intracellular calcium concentration). The quantitative measurement of leptin in serum was performed using a leptin enzyme immunoassay-ELISA kit (DRG Diagnostics, Marburg, Germany). Information prior to surgery was collected from medical records.

### 2.1. Statistical Analyses

For statistical analysis, Student's *t*-test was used to analyze the parametric data to compare the VSG and RYGB groups. For statistical purposes, comparisons were considered significant at *p* < 0.05. For statistical analysis of VSG before x VSG after and BGYR before x-BGYR after, one-way ANOVA was used, having as factors the type of surgery before and after the procedure. Tukey's post hoc test was used to observe differences between groups.

## 3. Results

### 3.1. BMI in VSG and RYGB Procedures

**Figure 1:**
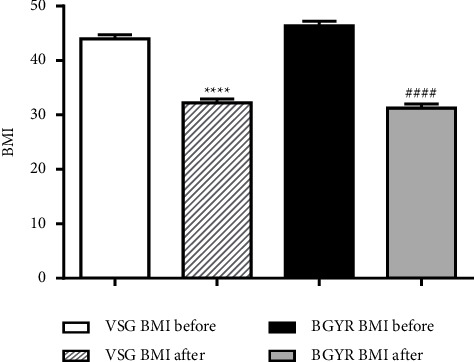
BMI before and after surgery, intragroup. Results are presented as mean ± SEM. Data were analyzed by one-way ANOVA followed by Tukey's test, ^*∗∗∗∗*^*p* < 0.001 VSG before × VSG after and ^####^*p* < 0.001 BGYR before × BGYR after. Groups: VSG before, *n* = 57; VSG after, *n* = 57; BGYR before, *n* = 58; BGYR after, *n* = 60. Vertical sleeve gastrectomy (VSG); RYGB: Roux-en-Y gastric bypass.

Baseline BMI values were not significantly different compared to those in the surgical procedures (VSG = 32.23 ± 0.70; RYGB = 31.29 ± 0.74; *p*=0.3590) (Table 1). Analysis of variance indicated a significant difference for both VSG and RYGB intragroups (*p* < 0.0001). Postsurgery reductions in VSG and RYGB were −11.80 kg/cm^2^ (95% CI = −14.53 to −9.07) −15.10 kg/cm^2^ (95% CI = −17.79 to −12.41), respectively. There were equivalent BMI values at the preoperative period (DM = 2.36 kg/cm^2^; 95% CI = −0.36 to 5.08) and anthropometric baseline (Figure 1). The intragroup was similar to the significant difference although there were no differences between postoperative groups' period (DM = −0.94 kg/cm^2^; 95% CI = −3.64 to 1.76) (Figure 1).

### 3.2. PTH in VSG and RYGB Procedures

Parathyroid hormone levels at baseline (PTH) were significantly different between procedures VSG (46.25 ± 3.40 ng/L) and RYGB (61.29 ± 3.98 ng/L) (*p*=0.006) (Table 2).

### 3.3. Total Calcium, Appendicular Skeletal Muscle Mass (ASMM), Total Skeletal Muscle Mass (Total SSM), Baumgartner Index (BAUM), Moderate and Vigorous IPAQ, Light IPAQ, and Body Fat Percentage (%BFP) in VSG and RYGB Procedures

There was no significant difference in the total calcium (ASMM), skeletal muscle mass (total SSM), the Baumgartner index (BAUM) (appendicular lean mass in relation to body surface) to indicate low muscle mass and sarcopenia, and the moderate and vigorous International Physical Activity Questionnaire (IPAQ and light IPAQ). The results are described below.

Total calcium (VSG = 9.15 ± 0.07 mg/dl; RYGB = 9.10 ± 0.011 mg/dl; *p*=0.708), ASMM (VSG = 20.33 ± 0.56 kg; RYGB = 19.61 ± 0.51 kg; *p*=0.342), Total SSM (VSG = 44.23 ± 1.0 kg; RYGB = 44.02 ± 1.09 kg; *p*=0.888), BAUM (VSG = 7.76 ± 0.17 kg; RYGB = 7.46 ± 0.16 kg; *p*=0.201), moderate and vigorous IPAQ (VSG = 247.1 ± 36.20; RYGB = 276.8 ± 31.37; *p*=0.535), light IPAQ (VSG = 189.6 ± 21.72; RYGB = 179.3 ± 26.92; *p*=0.767), and %BFP (VSG = 44.14 ± 0.97%; RYGB = 42.09 ± 1.33%; *p*=0.221) (Table 2).

### 3.4. Total BMD in VSG and RYGB Procedures

At baseline, total bone mineral density (BMD) was different between the group (*p*=0.005), in which the average of the VSG group was 1.24 ± 0.010 g/cm^2^, while the average of the RYGB group was 1.20 ± 0.010 g/cm^2^ (Table 2).

### 3.5. Plasma Insulin Concentration in VSG and RYGB Procedures

Insulin levels were significantly different between the groups (*p*=0.013); for VSG, it was 7.44 ± 0.57 (U/L) on average, while for RYGB, it was 5.60 ± 0.39 (U/L) average (Table 2).

### 3.6. Serum Levels of Vitamin D in VSG and RYGB Procedures

**Figure 2:**
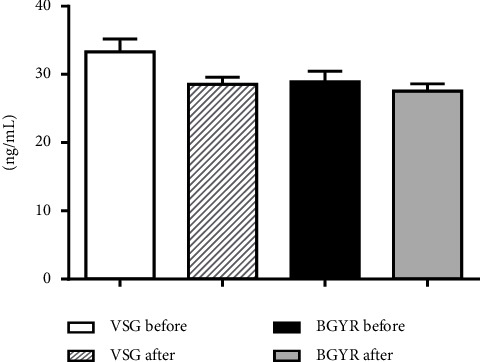
Vitamin D pre and postprocedure VSG and RYGB. Results are presented as mean ± SEM. Data were analyzed by one-way ANOVA followed by Tukey's test. Groups: VSG before, *n* = 24; VSG after, *n* = 50; BGYR before, *n* = 15; BGYR after, *n* = 54. Vertical sleeve gastrectomy (VSG); RYGB: Roux-en-Y gastric bypass.

There were no significant differences between vitamin D groups (25-hydroxyvitamin: D-25OHD), (VSG = 28.54 ± 1.03 ng/ml; RYGB = 27.58 ± 1.04 ng/ml; *p*=0.514) (Table 2). No other significant associations were found in the vitamin D intragroup (*p* > 0.05) in both surgical procedures (Figure 2).

## 4. Discussion

Analysis of BMI and vitamin D variables in the intragroup considering VSG were significantly different, while for the RYGB procedure, only BMI was significant. These results corroborate to show significant reduction in BMI after bariatric procedures, both VSG and RYGB procedures; however, for vitamin D, Corbeels et al. reported greater deficiency after RYGB-type procedures, compared to VSG; the latter also presents vitamin deficiency [4]. MI, total calcium, AMMI, total SSM, ASMM, moderate and vigorous IPAQ, light IPAQ, %BFP, and vitamin D showed no differences between the groups, while PTH, BMD, and insulin were significantly different.

Hewitt et al. observed that PTH increases after RYGB surgical procedures for obesity, with longitudinal studies indicating that this increase accentuates over time. Preoperative patients' serum 25-hydroxyvitamin D levels were below 50 nmol/L and also had higher PTH levels both in the preoperative and postoperative periods [5].

This study also confirmed the inverse relationship that exists between 25-hydroxyvitamin D and PTH levels. It was observed that an increase of 1 nmol/L in 25-hydroxyvitamin D was responsible for a decrease of 0.031 nmol/L of PTH during the postoperative period, monitoring of the operative surgery, reinforcing the relationship and importance of maintaining adequate levels of vitamin D for the regulation of thyroid hormones both before and after these procedures [5].

The same authors observed calcium reduction absorption after RYGB surgery but did not establish a clear relationship between calcium and PTH levels [5]. Calcium levels was also associated with vitamin D decrease, which is important to regulate the active transport of calcium, the predominant process of absorption of normal or low levels [4]. Borges et al. reported that, in RYGB-type surgeries, there is a marked reduction in calcium and vitamin D absorption, although the present study did not find significant differences for total calcium [16].

Bariatric surgeries impact bone metabolism, as weight loss, whether due to dietary restrictions, medications, or surgical procedures, is associated with a bone mineral density (BMD) decrease and bone turnover increase; these changes were higher in bariatric surgery. Comparisons between RYGB and VSG found a more pronounced bone loss in the RYGB type of procedure, mainly in the femoral neck and hip. BMD differences may be associated with hormonal patterns after the procedure, since hormones derived from fat and from the intestine interfere with bone health, as some studies have already shown [6]. The more accentuated bone loss in the RYGB procedure may also be associated with duodenal deviation, since it is in the duodenum where the increase in active calcium transport occurs, and the level of bone loss in this type of procedure is extended over the next 2 years postsurgical. The stomach volumetric reduction occurs in the VSG procedure; however, bone loss is lower [4].

Calcium and vitamin D deficiencies are the main factors that lead to accelerate lower bone, with a decrease in calcium levels with a 10% incidence and 25–73% prevalence of vitamin D, common before surgery [6]. The largest study carried out with morbidly obese patient candidates for bariatric surgery assessed nutritional and vitamin A deficiencies. It was found that 48% already had vitamin D deficiency before surgery [17].

Previously, vitamin deficiency was present in most obese patients, with vitamin D deficiency being the most prevalent, and it is known that this propensity occurs due to a volumetric dilution and sequestration of vitamin D by adipose tissue constituting a fat-soluble hormone. Adipose tissue needs more vitamin D to meet the requirements of this individual. In addition, studies have shown that 25-hydroxyvitamin D levels are also influenced by binding protein and vitamin D receptor functioning and that less functional receptors are present in obese individuals [6]. Other factors associated with vitamin D deficiency in the obese population are decreased synthesis through the skin and reduced liver production due to steatosis. In addition, a study showed that for every 1 kg/m^2^ increase in BMI, there was a reduction of 1.3 nmol/L of serum 25-hydroxyvitamin D [17]. They also found an inverse correlation between an increase in BMI and a decrease in serum concentration of 25-hydroxyvitamin D. One study investigated a total of 1,277 subjects with respect to various parameters such as body fat percentage, waist circumference, and waist-to-hip ratio. This study demonstrated a significant and inverse association between serum 25-hydroxyvitamin D levels, waist circumference, and waist-to-hip ratio, suggesting that obesity-associated phenotypes are closely linked to low levels of this vitamin [16].

Another cross-sectional study carried out on 340 obese individuals found a negative relationship between trunk fat and BMD, vitamin D, osteocalcin, IGF-1, insulin resistance, and inflammatory markers. An additional study performed on 859 random subjects suggested that increased levels of leptin and interleukin-6, which are mainly produced by adipose tissue, could inhibit 25-hydroxyvitamin D synthesis [16].

Another study with phosphocalcium metabolism in the VSG procedure showed that 95% of patients were vitamin D deficient before surgery and had increased levels of PTH. After surgery, vitamin D levels increased and PTH levels decreased. Another study that performed postsurgical evaluation in patients with previous higher levels of vitamin D found that there was no variation in PTH levels and that vitamin D levels also increased. When evaluating the RYGB procedure, postsurgical vitamin D deficiency rates were very high [17].

## 5. Conclusion

The analysis between groups had similar values in body composition and postsurgical vitamin D levels, with significant differences only for the PTH, BMD, and insulin variables, demonstrating that both procedures are effective in reducing fat mass. Regarding vitamin D, despite the similarity between groups, it is necessary to pay more attention to results described in the literature, which shows divergence, with RYGB responsible for the greatest levels of reduction. Both VSG and RYGB lead to similar changes in fat mass without increases in 25-OH-vitamin D.

Despite PTH and BMD postsurgery was significant different between groups, the active calcium transport did not decrease (transport occurs RYGB procedure included duodenal diversion of active calcium). PTH for RYGB increased, as would be expected due to accentuated decrease in vitamin D levels in this type of procedure as described in the literature.

Although bariatric surgery benefits obese patients, minimizing the risks of comorbidities and decreasing mortality rates, it is necessary to pay attention to nutritional and vitamin deficiencies, especially vitamin D, resulting from these procedures, which can aggravate or even develop secondary problems.

## Figures and Tables

**Table 1:** Anthropometric assessment of patients who underwent VSG or RYGB surgery, 2021.

Variable	VSG	BGYR	*p* value
BMI	32.23 ± 0.7 (*n* = 57)	31.29 ± 0.74 (*n* = 60)	0.359
Baseline weight	114.14 ± 1.79 (*n* = 57)	122.76 ± 2.65 (*n* = 60)	**0.009** ^*∗*^
Postoperative weight	83.58 ± 1.88 (*n* = 57)	82.25 ± 1.93 (*n* = 60)	0.623
Height	1.61 ± 0.01 (*n* = 57)	1.62 ± 0.01 (*n* = 60)	0.481
Lowest weight	74.99 ± 1.62 (*n* = 57)	74.22 ± 1.84 (*n* = 60)	0.755
Time to lowest weight (years)	1.25 ± 0.12 (*n* = 57)	1.51 ± 0.17 (*n* = 60)	0.223
% weight lost	34.05 ± 1.17 (*n* = 57)	39.02 ± 1.32 (*n* = 60)	**0.006** ^*∗*^
AC	98.20 ± 1.54 (*n* = 57)	94.05 ± 1.75 (*n* = 60)	0.079
NC	35.02 ± 0.39 (*n* = 57)	33.81 ± 0.42 (*n* = 60)	**0.037** ^*∗*^
Weight gain (kg)	9.60 ± 1.31 (*n* = 51)	9.09 ± 1.15 (*n* = 53)	0.770
Weight regain (%)	23.24 ± 2.81 (*n* = 51)	19.09 ± 2.28 (*n* = 53)	0.252

Results are presented as means ± SEM. Data were analyzed by an unpaired two-tailed student's *t*-test. ^*∗*^*p* < 0.05 was accepted significance. Vertical sleeve gastrectomy (VSG); RYGB: Roux-en-Y gastric bypass BMI: body mass index; AC: abdominal circumference; NC: neck circumference. Significance in bold with asterisk.

**Table 2:** Metabolic and biochemical evaluation of patients undergoing sleeve or RYGB surgery, 2021.

Variables	VSG	BGYR	*p* value
PTH	46.25 ± 3.40 (*n* = 47)	61.29 ± 3.98 (*n* = 55)	**0.006** ^*∗*^
Total calcium	9.15 ± 0.07 (*n* = 52)	9.10 ± 0.11 (*n* = 57)	0.708
Corrected calcium	9.79 ± 0.08 (*n* = 52)	9.88 ± 0.06 (*n* = 57)	0.365
ASMM	20.33 ± 0.51 (*n* = 53)	19.61 ± 0.51 (*n* = 58)	0.343
Total SMM	44.23 ± 1.00 (*n* = 53)	44.02 ± 1.09 (*n* = 58)	0.888
BAUM	7.76 ± 0.17 (*n* = 53)	7.46 ± 0.16 (*n* = 58)	0.201
IPAQ moderate	247.1 ± 36.20 (*n* = 55)	276.8 ± 31.37 (*n* = 60)	0.534
IPAQ light	189.6 ± 21.72(*n* = 55)	179.3 ± 26.92 (*n* = 60)	0.767
%BF	44.14 ± 0.97 (*n* = 54)	42.09 ± 1.33 (*n* = 58)	0.221
Total BMD	1.24 ± 0.01 (*n* = 54)	1.20 ± 0.01 (*n* = 58)	**0.0056** ^*∗*^
Insulin	7.44 ± 0.57 (*n* = 52)	5.60 ± 0.39 (*n* = 42)	**0.013** ^*∗*^
Vit D-25OHD	28.54 ± 1.03 (*n* = 50)	27.58 ± 1.04 (*n* = 54)	0.514

Results are presented as means ± SEM. Data were analyzed by an unpaired two-tailed student's *t*-test. ^*∗*^*p* < 0.05 was accepted significance. Vertical sleeve gastrectomy (VSG); RYGB: Roux-en-Y gastric bypass; PTH: parathyroid hormone; ASMM: appendicular skeletal muscle mass; Total SMM: skeletal muscle mass; BAUM: Baumgartner index; IPAQ: International Physical Activity Questionnaire; %BF: body fat; BMD: bone mineral density; 25OHD: 25-hydroxyvitamin D. Significance in bold with asterisk.

## Data Availability

Available data can be made public, in accordance with the journal's policy. All statistical analysis was performed using GraphPadPrism 6.0.
